# 4-[(2′-Cyano­biphenyl-4-yl)meth­yl]morpholin-4-ium perchlorate

**DOI:** 10.1107/S1600536812012792

**Published:** 2012-03-31

**Authors:** Hua-Yong Ou-Yang

**Affiliations:** a Department of Chemical Engineering, Nanjing College of Chemical Technology, Nanjing 210048, People’s Republic of China

## Abstract

In the title salt, C_18_H_19_N_2_O^+^·ClO_4_^−^, the morpholinium ring adopts a chair conformation, while the two benzene rings make a dihedral angle of 62.65 (17)°. Inter­molecular N—H⋯N hydrogen bonds and weak C—H⋯O inter­actions occur in the crystal structure.

## Related literature

The title complound was investigated as part of a search for dielectric ferroelectric materials. For background to ferroelectric materials, see: Haertling (1999 ▶); Homes *et al.* (2001 ▶).
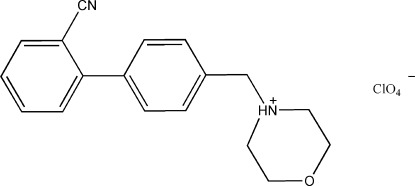


## Experimental

### 

#### Crystal data

C_18_H_19_N_2_O^+^·ClO_4_^−^
*M* *_r_* = 378.80
Monoclinic, 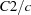

*a* = 22.997 (5) Å
*b* = 10.679 (2) Å
*c* = 14.899 (3) Å
β = 92.96 (3)°
*V* = 3654.2 (13) Å^3^
*Z* = 8
Mo *K*α radiation
μ = 0.24 mm^−1^
*T* = 293 K
0.20 × 0.20 × 0.20 mm

#### Data collection

Rigaku Mercury2 diffractometer
18531 measured reflections
4191 independent reflections
2478 reflections with *I* > 2σ(*I*)
*R*_int_ = 0.066

#### Refinement

*R*[*F* ^2^ > 2σ(*F* ^2^)] = 0.070
*wR*(*F* ^2^) = 0.202
*S* = 1.04
4191 reflections
235 parameters
1 restraint
H-atom parameters constrained
Δρ_max_ = 0.59 e Å^−3^
Δρ_min_ = −0.34 e Å^−3^

### 

Data collection: *CrystalClear* (Rigaku, 2005 ▶); cell refinement: *CrystalClear*; data reduction: *CrystalClear*; program(s) used to solve structure: *SHELXTL* (Sheldrick, 2008 ▶); program(s) used to refine structure: *SHELXTL*; molecular graphics: *SHELXTL*; software used to prepare material for publication: *SHELXTL*.

## Supplementary Material

Crystal structure: contains datablock(s) I, global. DOI: 10.1107/S1600536812012792/xu5487sup1.cif

Structure factors: contains datablock(s) I. DOI: 10.1107/S1600536812012792/xu5487Isup2.hkl

Supplementary material file. DOI: 10.1107/S1600536812012792/xu5487Isup3.cml

Additional supplementary materials:  crystallographic information; 3D view; checkCIF report

## Figures and Tables

**Table 1:** Hydrogen-bond geometry (Å, °)

*D*—H⋯*A*	*D*—H	H⋯*A*	*D*⋯*A*	*D*—H⋯*A*
N1—H1⋯N2^i^	0.82	2.12	2.921 (4)	166
C2—H2*B*⋯O4^ii^	0.97	2.57	3.255 (5)	128
C5—H5*B*⋯O4^iii^	0.97	2.59	3.515 (6)	161
C5—H5*C*⋯O4^iv^	0.97	2.57	3.474 (7)	154

Symmetry codes: (i) 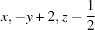; (ii) 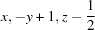; (iii) 
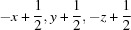
; (iv) 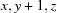.

**Fig. 1.**
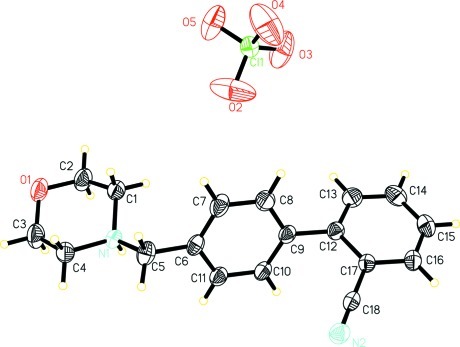
Perspective structure of the title compound, showing the atom-numbering scheme. Displacement ellipsoids are drawn at the 30% probability level.

**Fig. 2.**
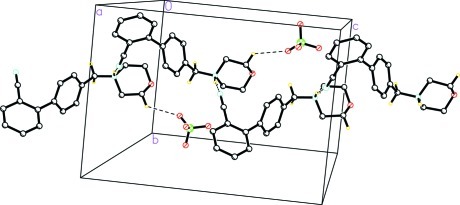
The crystal packing of the title compound viewed along the a axis showing the hydrogen bondings network.

## supplementary crystallographic information

### Comment

At present, much attention in ferroelectric material field is focused on developing ferroelectric pure organic or inorganic compounds (Haertling, 1999; Homes *et al.*, 2001). In order to find more dielectric ferroelectric materials, we investigate the physical properties of the title compound. Here we report the synthesis and crystal structure of the title compound, 4-((2'-cyanobiphenyl-4-yl)methyl)morpholin-4-ium perchlorate (Fig. 1).

The bond distances and bond angles in the title compound agree very well with the corresponding distances and angles reported for a closely related compound. In this structure, the intermolecular N–H···N and C–H···O hydrogen bonds link the cations and anions to chains (Table 1). The dihedral angle between the benzene rings in the cation is 62.65 (17).

### Experimental

To a stirred solution of 4'-(morpholinomethyl)biphenyl-2-carbonitrile (5.56 g, 0.02 mol) in 30 mL of methanol, perchloric acid (2.87 g, 0.02 mol) was added at the room temperature. The precipitate was filtered and washed with a small amount of ethanol 95%. Single crystals suitable for X-ray diffraction analysis were obtained from slow evaporation of a solution of the title compound in water at room temperature.

### Refinement

The H atoms were positioned geometrically and refined using a riding model, with N—H = 0.82 and C—H = 0.93–0.96 Å, *U*_iso_(H) = 1.2*U*_eq_(C) and 1.5U_eq_(N).

### Crystal data

**Table:**

C_18_H_19_N_2_O^+^·ClO_4_^−^	*F*(000) = 1584
*M**_r_* = 378.80	*D*_x_ = 1.377 Mg m^−^^3^
Monoclinic, *C*2/*c*	Mo *K*α radiation, λ = 0.71073 Å
Hall symbol: -C 2yc	Cell parameters from 4191 reflections
*a* = 22.997 (5) Å	θ = 2.6–27.5°
*b* = 10.679 (2) Å	µ = 0.24 mm^−^^1^
*c* = 14.899 (3) Å	*T* = 293 K
β = 92.96 (3)°	Prism, colorless
*V* = 3654.2 (13) Å^3^	0.20 × 0.20 × 0.20 mm
*Z* = 8	

### Data collection

**Table:**

Rigaku Mercury2 diffractometer	2478 reflections with *I* > 2σ(*I*)
Radiation source: fine-focus sealed tube	*R*_int_ = 0.066
Graphite monochromator	θ_max_ = 27.5°, θ_min_ = 3.2°
Detector resolution: 13.6612 pixels mm^-1^	*h* = −29→29
CCD_Profile_fitting scans	*k* = −13→13
18531 measured reflections	*l* = −19→19
4191 independent reflections	

### Refinement

**Table:**

Refinement on *F*^2^	Primary atom site location: structure-invariant direct methods
Least-squares matrix: full	Secondary atom site location: difference Fourier map
*R*[*F*^2^ > 2σ(*F*^2^)] = 0.070	Hydrogen site location: inferred from neighbouring sites
*wR*(*F*^2^) = 0.202	H-atom parameters constrained
*S* = 1.04	*w* = 1/[σ^2^(*F*_o_^2^) + (0.0836*P*)^2^ + 4.9367*P*] where *P* = (*F*_o_^2^ + 2*F*_c_^2^)/3
4191 reflections	(Δ/σ)_max_ < 0.001
235 parameters	Δρ_max_ = 0.59 e Å^−^^3^
1 restraint	Δρ_min_ = −0.34 e Å^−^^3^

### Special details

**Table:**

Geometry. All e.s.d.'s (except the e.s.d. in the dihedral angle between two l.s. planes) are estimated using the full covariance matrix. The cell e.s.d.'s are taken into account individually in the estimation of e.s.d.'s in distances, angles and torsion angles; correlations between e.s.d.'s in cell parameters are only used when they are defined by crystal symmetry. An approximate (isotropic) treatment of cell e.s.d.'s is used for estimating e.s.d.'s involving l.s. planes.
Refinement. Refinement of *F*^2^ against ALL reflections. The weighted *R*-factor *wR* and goodness of fit *S* are based on *F*^2^, conventional *R*-factors *R* are based on *F*, with *F* set to zero for negative *F*^2^. The threshold expression of *F*^2^ > σ(*F*^2^) is used only for calculating *R*-factors(gt) *etc*. and is not relevant to the choice of reflections for refinement. *R*-factors based on *F*^2^ are statistically about twice as large as those based on *F*, and *R*- factors based on ALL data will be even larger.

### Fractional atomic coordinates and isotropic or equivalent isotropic displacement parameters (Å^2^)

**Table:**

	*x*	*y*	*z*	*U*_iso_*/*U*_eq_	
Cl1	0.14467 (4)	0.33074 (8)	0.21821 (6)	0.0575 (3)	
O2	0.15678 (19)	0.4547 (4)	0.2333 (4)	0.169 (2)	
O3	0.08570 (14)	0.3054 (5)	0.2221 (3)	0.1361 (16)	
O4	0.1785 (2)	0.2616 (5)	0.2795 (3)	0.165 (2)	
O5	0.16055 (18)	0.3058 (5)	0.1321 (3)	0.1407 (16)	
N1	0.18775 (11)	0.9619 (2)	0.12783 (16)	0.0469 (6)	
H1	0.1571	0.9991	0.1172	0.070*	
C11	0.09773 (15)	0.9566 (3)	0.2946 (2)	0.0544 (8)	
H11A	0.0957	1.0410	0.2796	0.065*	
C9	0.05497 (14)	0.7774 (3)	0.3637 (2)	0.0491 (8)	
C12	0.01047 (14)	0.7227 (3)	0.4209 (2)	0.0484 (8)	
C18	0.04350 (14)	0.8644 (3)	0.5429 (2)	0.0481 (8)	
N2	0.07272 (13)	0.9427 (3)	0.57063 (19)	0.0621 (8)	
C6	0.14489 (14)	0.8859 (3)	0.27003 (19)	0.0511 (8)	
C17	0.00559 (13)	0.7666 (3)	0.5091 (2)	0.0456 (7)	
O1	0.22810 (13)	0.9243 (3)	−0.04846 (17)	0.0813 (9)	
C1	0.18162 (17)	0.8401 (3)	0.0800 (2)	0.0637 (10)	
H1A	0.1465	0.7981	0.0974	0.076*	
H1B	0.2146	0.7870	0.0966	0.076*	
C16	−0.03494 (14)	0.7160 (3)	0.5651 (2)	0.0561 (8)	
H16A	−0.0378	0.7466	0.6232	0.067*	
C10	0.05393 (15)	0.9032 (3)	0.3408 (2)	0.0529 (8)	
H10A	0.0229	0.9526	0.3571	0.063*	
C7	0.14461 (17)	0.7581 (4)	0.2892 (2)	0.0691 (11)	
H7A	0.1749	0.7079	0.2710	0.083*	
C4	0.23714 (16)	1.0349 (4)	0.0925 (2)	0.0612 (9)	
H4B	0.2737	0.9941	0.1100	0.073*	
H4C	0.2378	1.1183	0.1183	0.073*	
C8	0.10025 (18)	0.7053 (4)	0.3347 (3)	0.0687 (11)	
H8A	0.1007	0.6197	0.3461	0.082*	
C13	−0.02633 (18)	0.6260 (4)	0.3924 (3)	0.0658 (10)	
H13A	−0.0239	0.5939	0.3347	0.079*	
C5	0.19543 (15)	0.9467 (4)	0.2283 (2)	0.0598 (9)	
H5B	0.2300	0.8969	0.2421	0.072*	
H5C	0.2017	1.0286	0.2553	0.072*	
C2	0.17854 (19)	0.8610 (4)	−0.0204 (3)	0.0775 (12)	
H2B	0.1753	0.7808	−0.0508	0.093*	
H2C	0.1440	0.9095	−0.0373	0.093*	
C3	0.23077 (19)	1.0439 (4)	−0.0083 (3)	0.0776 (12)	
H3B	0.1956	1.0901	−0.0254	0.093*	
H3C	0.2636	1.0897	−0.0302	0.093*	
C15	−0.07050 (16)	0.6209 (4)	0.5342 (3)	0.0669 (10)	
H15A	−0.0974	0.5861	0.5714	0.080*	
C14	−0.06639 (18)	0.5771 (4)	0.4484 (3)	0.0739 (11)	
H14A	−0.0910	0.5132	0.4276	0.089*	

### Atomic displacement parameters (Å^2^)

**Table:**

	*U*^11^	*U*^22^	*U*^33^	*U*^12^	*U*^13^	*U*^23^
Cl1	0.0575 (5)	0.0613 (5)	0.0536 (5)	−0.0065 (4)	0.0016 (4)	0.0024 (4)
O2	0.138 (4)	0.073 (2)	0.288 (6)	0.009 (2)	−0.070 (4)	−0.045 (3)
O3	0.070 (2)	0.234 (5)	0.105 (3)	−0.045 (3)	0.0218 (18)	−0.030 (3)
O4	0.152 (4)	0.166 (4)	0.172 (4)	−0.017 (3)	−0.058 (3)	0.105 (4)
O5	0.131 (3)	0.204 (5)	0.093 (3)	−0.004 (3)	0.050 (2)	−0.013 (3)
N1	0.0430 (14)	0.0590 (16)	0.0390 (13)	0.0028 (12)	0.0041 (11)	0.0011 (12)
C11	0.065 (2)	0.057 (2)	0.0423 (17)	0.0102 (17)	0.0091 (15)	0.0034 (15)
C9	0.0586 (19)	0.0542 (19)	0.0345 (15)	0.0071 (16)	0.0033 (14)	0.0018 (14)
C12	0.0531 (18)	0.0508 (18)	0.0410 (17)	0.0039 (15)	0.0011 (14)	0.0037 (14)
C18	0.0490 (19)	0.058 (2)	0.0377 (16)	0.0062 (16)	0.0078 (14)	0.0009 (15)
N2	0.0631 (19)	0.070 (2)	0.0531 (17)	−0.0088 (16)	0.0029 (14)	−0.0086 (15)
C6	0.0535 (19)	0.070 (2)	0.0296 (15)	0.0066 (16)	0.0025 (13)	0.0023 (15)
C17	0.0445 (16)	0.0491 (18)	0.0435 (17)	0.0022 (14)	0.0039 (13)	0.0038 (14)
O1	0.088 (2)	0.102 (2)	0.0570 (16)	−0.0190 (17)	0.0321 (14)	−0.0119 (15)
C1	0.067 (2)	0.068 (2)	0.057 (2)	−0.0121 (19)	0.0167 (18)	−0.0106 (18)
C16	0.0545 (19)	0.061 (2)	0.054 (2)	0.0009 (17)	0.0109 (16)	0.0085 (17)
C10	0.059 (2)	0.059 (2)	0.0415 (17)	0.0151 (16)	0.0104 (15)	0.0036 (15)
C7	0.071 (2)	0.076 (3)	0.062 (2)	0.031 (2)	0.0225 (19)	0.018 (2)
C4	0.061 (2)	0.065 (2)	0.058 (2)	−0.0091 (18)	0.0111 (17)	−0.0017 (18)
C8	0.087 (3)	0.057 (2)	0.064 (2)	0.021 (2)	0.021 (2)	0.0148 (18)
C13	0.079 (3)	0.066 (2)	0.052 (2)	−0.010 (2)	−0.0044 (19)	−0.0075 (18)
C5	0.054 (2)	0.089 (3)	0.0355 (16)	−0.0001 (18)	−0.0010 (14)	0.0057 (17)
C2	0.082 (3)	0.101 (3)	0.051 (2)	−0.023 (2)	0.019 (2)	−0.020 (2)
C3	0.084 (3)	0.094 (3)	0.056 (2)	−0.013 (2)	0.019 (2)	0.012 (2)
C15	0.058 (2)	0.068 (2)	0.076 (3)	−0.0089 (19)	0.0103 (19)	0.015 (2)
C14	0.073 (3)	0.061 (2)	0.087 (3)	−0.018 (2)	−0.004 (2)	0.001 (2)

### Geometric parameters (Å, º)

**Table:**

Cl1—O2	1.369 (4)	C1—C2	1.511 (5)
Cl1—O5	1.377 (4)	C1—H1A	0.9700
Cl1—O4	1.383 (4)	C1—H1B	0.9700
Cl1—O3	1.387 (3)	C16—C15	1.369 (5)
N1—C1	1.486 (4)	C16—H16A	0.9300
N1—C4	1.495 (4)	C10—H10A	0.9300
N1—C5	1.507 (4)	C7—C8	1.374 (5)
N1—H1	0.8180	C7—H7A	0.9300
C11—C10	1.373 (5)	C4—C3	1.505 (5)
C11—C6	1.385 (4)	C4—H4B	0.9700
C11—H11A	0.9300	C4—H4C	0.9700
C9—C8	1.382 (5)	C8—H8A	0.9300
C9—C10	1.386 (5)	C13—C14	1.378 (5)
C9—C12	1.485 (4)	C13—H13A	0.9300
C12—C13	1.388 (5)	C5—H5B	0.9700
C12—C17	1.404 (4)	C5—H5C	0.9700
C18—N2	1.136 (4)	C2—H2B	0.9700
C18—C17	1.435 (5)	C2—H2C	0.9700
C6—C7	1.395 (5)	C3—H3B	0.9700
C6—C5	1.494 (5)	C3—H3C	0.9700
C17—C16	1.393 (4)	C15—C14	1.369 (5)
O1—C2	1.407 (5)	C15—H15A	0.9300
O1—C3	1.411 (5)	C14—H14A	0.9300
			
O2—Cl1—O5	106.2 (3)	C9—C10—H10A	119.3
O2—Cl1—O4	107.8 (3)	C8—C7—C6	120.9 (3)
O5—Cl1—O4	110.1 (3)	C8—C7—H7A	119.5
O2—Cl1—O3	111.9 (3)	C6—C7—H7A	119.5
O5—Cl1—O3	107.9 (3)	N1—C4—C3	110.4 (3)
O4—Cl1—O3	112.8 (3)	N1—C4—H4B	109.6
C1—N1—C4	110.0 (3)	C3—C4—H4B	109.6
C1—N1—C5	112.7 (3)	N1—C4—H4C	109.6
C4—N1—C5	110.7 (3)	C3—C4—H4C	109.6
C1—N1—H1	105.8	H4B—C4—H4C	108.1
C4—N1—H1	109.9	C7—C8—C9	121.1 (3)
C5—N1—H1	107.5	C7—C8—H8A	119.5
C10—C11—C6	120.7 (3)	C9—C8—H8A	119.5
C10—C11—H11A	119.6	C14—C13—C12	120.8 (4)
C6—C11—H11A	119.6	C14—C13—H13A	119.6
C8—C9—C10	117.8 (3)	C12—C13—H13A	119.6
C8—C9—C12	120.9 (3)	C6—C5—N1	114.0 (3)
C10—C9—C12	121.3 (3)	C6—C5—H5B	108.8
C13—C12—C17	117.3 (3)	N1—C5—H5B	108.8
C13—C12—C9	122.9 (3)	C6—C5—H5C	108.8
C17—C12—C9	119.8 (3)	N1—C5—H5C	108.8
N2—C18—C17	178.8 (4)	H5B—C5—H5C	107.7
C11—C6—C7	117.8 (3)	O1—C2—C1	111.5 (3)
C11—C6—C5	120.6 (3)	O1—C2—H2B	109.3
C7—C6—C5	121.5 (3)	C1—C2—H2B	109.3
C16—C17—C12	121.3 (3)	O1—C2—H2C	109.3
C16—C17—C18	119.0 (3)	C1—C2—H2C	109.3
C12—C17—C18	119.7 (3)	H2B—C2—H2C	108.0
C2—O1—C3	109.1 (3)	O1—C3—C4	111.4 (3)
N1—C1—C2	110.2 (3)	O1—C3—H3B	109.3
N1—C1—H1A	109.6	C4—C3—H3B	109.3
C2—C1—H1A	109.6	O1—C3—H3C	109.3
N1—C1—H1B	109.6	C4—C3—H3C	109.3
C2—C1—H1B	109.6	H3B—C3—H3C	108.0
H1A—C1—H1B	108.1	C14—C15—C16	120.0 (4)
C15—C16—C17	119.5 (3)	C14—C15—H15A	120.0
C15—C16—H16A	120.3	C16—C15—H15A	120.0
C17—C16—H16A	120.3	C15—C14—C13	121.1 (4)
C11—C10—C9	121.5 (3)	C15—C14—H14A	119.4
C11—C10—H10A	119.3	C13—C14—H14A	119.4
			
C8—C9—C12—C13	−62.6 (5)	C5—C6—C7—C8	174.2 (3)
C10—C9—C12—C13	120.2 (4)	C1—N1—C4—C3	51.9 (4)
C8—C9—C12—C17	115.6 (4)	C5—N1—C4—C3	177.1 (3)
C10—C9—C12—C17	−61.7 (4)	C6—C7—C8—C9	−0.8 (6)
C10—C11—C6—C7	3.6 (5)	C10—C9—C8—C7	3.8 (6)
C10—C11—C6—C5	−173.6 (3)	C12—C9—C8—C7	−173.6 (3)
C13—C12—C17—C16	−0.6 (5)	C17—C12—C13—C14	0.9 (5)
C9—C12—C17—C16	−178.9 (3)	C9—C12—C13—C14	179.0 (4)
C13—C12—C17—C18	178.5 (3)	C11—C6—C5—N1	−85.9 (4)
C9—C12—C17—C18	0.3 (4)	C7—C6—C5—N1	97.0 (4)
N2—C18—C17—C16	−35 (18)	C1—N1—C5—C6	−62.2 (4)
N2—C18—C17—C12	146 (18)	C4—N1—C5—C6	174.2 (3)
C4—N1—C1—C2	−51.8 (4)	C3—O1—C2—C1	−62.5 (5)
C5—N1—C1—C2	−175.8 (3)	N1—C1—C2—O1	58.0 (5)
C12—C17—C16—C15	0.5 (5)	C2—O1—C3—C4	62.4 (4)
C18—C17—C16—C15	−178.7 (3)	N1—C4—C3—O1	−57.6 (4)
C6—C11—C10—C9	−0.6 (5)	C17—C16—C15—C14	−0.6 (6)
C8—C9—C10—C11	−3.1 (5)	C16—C15—C14—C13	0.8 (6)
C12—C9—C10—C11	174.2 (3)	C12—C13—C14—C15	−1.0 (6)
C11—C6—C7—C8	−2.9 (5)		

### Hydrogen-bond geometry (Å, º)

**Table:**

*D*—H···*A*	*D*—H	H···*A*	*D*···*A*	*D*—H···*A*
N1—H1···N2^i^	0.82	2.12	2.921 (4)	166
C2—H2*B*···O4^ii^	0.97	2.57	3.255 (5)	128
C5—H5*B*···O4^iii^	0.97	2.59	3.515 (6)	161
C5—H5*C*···O4^iv^	0.97	2.57	3.474 (7)	154

Symmetry codes: (i) *x*, −*y*+2, *z*−1/2; (ii) *x*, −*y*+1, *z*−1/2; (iii) −*x*+1/2, *y*+1/2, −*z*+1/2; (iv) *x*, *y*+1, *z*.
